# Visual analytics for concept exploration in subspaces of patient groups: Making sense of complex datasets with the Doctor-in-the-loop

**DOI:** 10.1007/s40708-016-0043-5

**Published:** 2016-03-21

**Authors:** Michael Hund, Dominic Böhm, Werner Sturm, Michael Sedlmair, Tobias Schreck, Torsten Ullrich, Daniel A. Keim, Ljiljana Majnaric, Andreas Holzinger

**Affiliations:** 1 Department of Computer and Information Science, University of Konstanz, Box 78, 78457 Konstanz, Germany; 2 University of Vienna, Vienna, Austria; 3 Graz University of Technology, Graz, Austria; 4 Frauenhofer Austria Research GmbH, Graz, Austria; 5 University of Konstanz, Konstanz, Germany; 6 Faculty of Medicine, JJ Strossmayer University of Osijek, Osijek, Croatia; 7 Research Unit HCI-KDD, Institute for Medical Informatics, Statistics and Documentation, Medical University Graz, Graz, Austria

**Keywords:** Knowledge discovery and exploration, Visual analytics, Subspace clustering, Subspace analysis, Subspace exploration and comparison

## Abstract

Medical doctors and researchers in bio-medicine are increasingly confronted with complex patient data, posing new and difficult analysis challenges. These data are often comprising *high-dimensional* descriptions of patient conditions and measurements on the success of certain therapies. An important analysis question in such data is to compare and correlate patient conditions and therapy results along with combinations of dimensions. As the number of dimensions is often very large, one needs to map them to a smaller number of relevant dimensions to be more amenable for expert analysis. This is because irrelevant, redundant, and conflicting dimensions can negatively affect effectiveness and efficiency of the analytic process (the so-called *curse of dimensionality*). However, the possible mappings from high- to low-dimensional spaces are ambiguous. For example, the similarity between patients may change by considering different combinations of relevant dimensions (*subspaces*). We demonstrate the potential of subspace analysis for the interpretation of high-dimensional medical data. Specifically, we present SubVIS, an interactive tool to visually explore subspace clusters from different perspectives, introduce a novel analysis workflow, and discuss future directions for high-dimensional (medical) data analysis and its visual exploration. We apply the presented workflow to a real-world dataset from the medical domain and show its usefulness with a domain expert evaluation.

## Introduction

Today, experts in medicine, biology, and the life sciences are not only confronted with increasingly large, but also complex and high-dimensional data. This situation will become even more pronounced with the ongoing trend towards personalized medicine [1]. One of the grand future challenges of biomedical informatics research is to gain knowledge from complex high-dimensional datasets [2]. Within such data, relevant and interesting *structural* and/or *temporal* patterns (“knowledge”) are often hidden and not accessible to domain experts. The concepts of “relevant” and “interesting,” however, are not crisply defined and are inherently dependent on the context and subjective judgment of investigators.

In this paper, we specifically focus on the challenges stemming from the high dimensionality often encountered in biomedical datasets. Examples range from longitudinal Rheumatology datasets, in which cohorts of patients are attributed with vectors in ${{\mathbb {R}}^{100}}$, up to DNA micro-array or protein data with a potentially arbitrary number of dimensions. Viewing, understanding, and effectively using such datasets pose many challenges to analysts.

Two typical analysis tasks of such data include understanding *similarities* between data records and *correlating* records with certain dimensions, some of which may be class-labeled data. In Data Mining, many tools have been proposed to support such analytic questions. For example, *clustering algorithms* search for groups of similar records, and *classification algorithms* find data structures to predict the class label of a previously unseen data record according to annotated (classified) training data. While many data analysis tools are known to date, their effective usage in practical application poses challenges which need to be overcome. In this paper, we focus on two such important challenges. The first relates to the high-dimensional nature of the data at hand (curse of dimensionality), and the second relates to the problem of interactively exploring and understanding the outcome of the automatic data analysis.

**Fig. 1:**
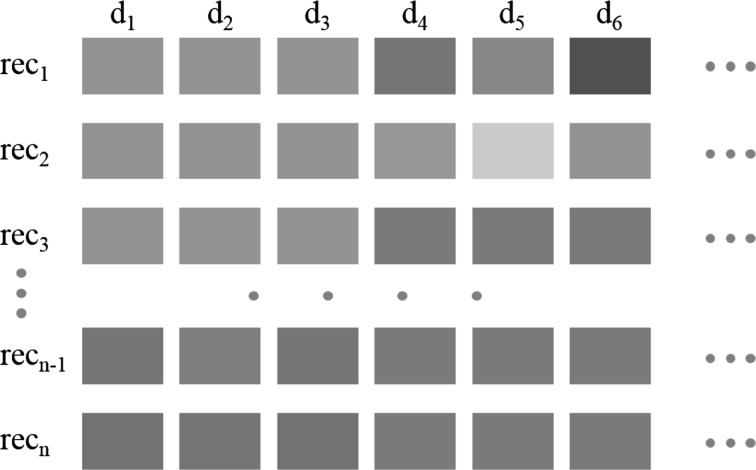
Effects in high-dimensional data: some objects are only similar in a subset of dimensions (=subspace), e.g., $rec_1,\,rec_2,\,rec_3$ in the first three and $rec_3,\,rec_{n-1},\,rec_n$ in the last three dimensions. Similar values in specific dimensions are encoded by the *same color*. (Color figure online)

The curse of dimensionality [3, 4] describes the fact that with increasing dimensionality the spanned volume of the data space increases so fast that the available data become sparse. This effect makes judging distances and finding meaningful similarity relationships hard. In fact, the concept of distance becomes less applicable as the number of dimensions grows, since the distance between any two points in a given dataset converges (concentration effects). Hence, traditional global approaches taking all dimensions into account become increasingly insufficient (**full-space** approaches). Instead, interesting patterns such as clusters might only exist in **subspaces** and remain hidden with traditional full-space approaches, disguised by the many potential *irrelevant* dimensions. Figure 1 illustrates the problem. To overcome these problems, subspace analysis techniques have been proposed which apply dimensionality reduction as an integral part of the data analysis. Subspace analysis techniques search for various relevant patterns in different subspaces of the original data, such as *subspace clustering* [5], or *subspace nearest neighbor search* [6].

While these are useful tools, interpretation of obtained results may be rather challenging for users, as the outcome may involve, e.g., large sets of subspace clusters, many of which contain redundant patterns, or patterns that are not relevant for a specific analysis goal. Therefore, also appropriate *visual-interactive presentation* and *exploration* of data and analysis results is needed. Techniques from Information Visualization [7] can help to provide effective overviews of data, to allow the user to change views on the fly, and hence to support discovery of relevant findings. Also, techniques from Visual Analytics [8, 9] which aim at involving the user closely in steering the analysis process and finding appropriate analysis parameters can be of help. Our vision is a “Doctor-in-the-Loop” system for biomedical experts that allows leveraging of state-of-the-art data analysis algorithms via intuitive visual interfaces, and fosters the communication of respective findings to interested stakeholders such as medical experts, patients, and their next-of-kin.

Here, we propose a Visual Analytics tool, *SubVIS*, to help explore biomedical patient data by combining *Sub*-space analysis algorithms with interactive *VIS*ualization. SubVIS provides an easy-to-use way to interact and analyze obtained subspaces from different perspectives. Furthermore, it helps to answer questions such as *what does it mean if a dimension occurs never/very often in different subspaces?* Using SubVIS, we present a case study on a real-world immunization dataset, illustrating the benefits of subspace over full-space analysis methods in finding possible explanations for a positive or negative vaccination treatment. We also propose an analysis workflow, which can help understand the relationship of patient clusters in context of attribute similarities and outcomes of the immunization treatment. The paper concludes with a discussion of limitations and possible extensions to subspace analysis applied in this domain.

## Background and related work

This section provides background information and relevant related work. We first discuss important *analytical approaches*, starting with the generic analysis problems of data clustering and feature selection, and the more specific tools for subspace analysis. We then briefly introduce *interactive and visual analysis techniques* for high-dimensional data in general and subspace analysis in particular. Finally, we discuss visualizations in the bio-medicine domain and delineate them from our approach.

### Data analysis approaches

#### Cluster analysis

Cluster analysis is a well-known tool to reduce large datasets to a smaller number of clusters which can be compared with each other and in relation to some target attribute of interest [10]. Parts of the existing methods can be distinguished into partition-based and density-based methods. Traditional (full-space) clustering approaches such as *k-means* or *hierarchical clustering* [10] take all dimensions into account. However, it has been shown that for many dimensions the so-called *curse of dimensionality* may prevent effective cluster analysis, as the similarity measure may become less discriminant [3, 4]. To this end, dimension reduction and subspace analysis techniques have been developed, as discussed next.

#### Dimension reduction and subspace analysis

There are several approaches to tackle the curse of dimensionality. Two of the most important techniques are *feature selection* and *feature extraction*. Often, these techniques are summarized under the umbrella term *dimension reduction*. The main goal of feature selection [11] is to remove irrelevant and redundant features. The relevance definition of a dimension usually depends either on a measurable quality criterion of the analysis (e.g., classification error) or based on an analysis independent measure of a single dimension such as the variance. By contrast, feature extraction or transformation techniques try to preserve the original structure of the data by transforming it into a lower-dimensional representation. Both Multidimensional Scaling (MDS) [12] and Principle Component Analysis (PCA) [13] are two well-known feature extraction techniques. MDS tries to preserve the pair-wise distance between all data objects and represent it typically in a 2D space. Likewise, PCA computes weighted linear combinations by preserving the variance in the data. Dimensionality reduction techniques aim at a single, lower-dimensional representation of the data, either by changing the structure of the data to improve the quality of an analysis or by representing the original structure with fewer dimensions. However, in many high-dimensional applications, there is not a single pattern, such as a single clustering result, in one subset of dimensions, but rather multiple different clusters in multiple different subspaces of the original data which leads to the aim of subspace analysis: detection of different patterns in different subspaces, leading to a large result set of patterns each associated with a single subspace.

**Fig. 2:**
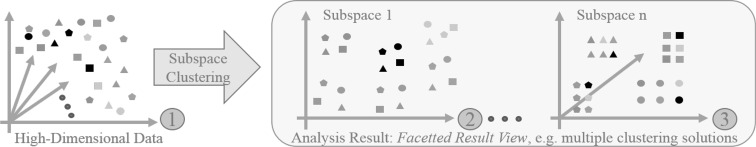
Subspace clustering: algorithms compute multiple, alternative solutions in different subspaces, i.e., clustering by *color* (subspace 1) or by shape (subspace n). (Color figure online)

#### Subspace clustering

The most well-known subspace analysis technique is *subspace clustering* which is illustrated in Fig. 2. Subspace clustering algorithms search for clusters not in the whole data space, but within different subsets of dimensions (called *subspaces*) in which discriminating clusters can be found [14]. The goal is to understand data in terms of (a) groups of similar records (clusters), and (b) the underlying relationship to the dimensions (subspaces). As an outcome of subspace cluster analysis applied on a high-dimensional data ①, depending on the parameterization and/or subspace cluster method, clusterings in multiple *different subspaces* may be found, constituting different notions of similarity, e.g., grouping according to color ① or shape ①. Each subspace cluster may give rise to a different interpretation. Subspace clustering methods may not only provide more meaningful clustering results for high-dimensional data, but also the information on the relevant dimension sets per cluster may provide valuable insight into the data. Depending on the underlying algorithm, we can state that all cluster members are similar to each other w.r.t. the dimensions of the subspace [10].

### Interactive and visual data exploration

Data analysis algorithms typically require parameters to be set, and often multiple solutions need to be considered before arriving at a satisfactory result. To this end, methods of interactive and visual exploration of the data and the analysis outputs can be very helpful [15].

#### Visualizing high-dimensional data

A number of visualization techniques have been developed for exploration of high-dimensional data and clusterings. For example, Parallel Coordinate Plots [16] map high-dimensional data to Polylines, allowing the user to discern groups in data and potentially relevant relationships, effective for moderate numbers of dimensions. Another standard approach is to reduce data dimensionality and show relationships of data points by their positions in a data projection [17]. Seo and Shneiderman [18] proposed an approach to visually compare data clusterings with constituent data dimensions. Other approaches for comparative visualization of subspaces are based on induced similarity hierarchies [19] or on 2D cluster projection [20]. The latter approaches allow to compare how data structures change across different feature spaces.

#### Visualizations for subspace clusters

The most challenging aspect of subspace clustering algorithms is the interpretation of the results. On one hand, the understanding of compact clusters in subsets of dimensions is challenging itself. A user might ask why a specific subspace has been chosen by the algorithm and why specific dimensions are ignored in a specific subspace. On the other hand, subspace clustering algorithms usually produce a large set of subspaces, many of which represent redundant clusters, not mentioning that different parameter settings may result in different or even more redundant clusters. Hereby, redundancy can be seen either from a dimension, or from a cluster member perspective. Therefore, there is a need for visualizations that support the user in finding good parameter settings, but also to extract subspaces and subspace clusters that are relevant for a specific application.

Some of the first tools for the comparison of subspace cluster results are VISA [21] and Morpheus [22], which implement a simple glyph alternative to represent and compare subspace clusters. CoDA [23] and MCExplorer [24] are two approaches to identify groups of subspace clusters that are similar to each other. Both tools use novel similarity measures to compute the similarity of subspace clusters based on a combination of its cluster members and dimensions. Additionally, the tools provide interactive visualizations to analyze and adapt concepts of subspace cluster groups.

A more recent tool called ClustNails [25] proposes to explore subspace clusters by a combination of a heatmap for similar values of the dimensions, and a glyph representation to explore similarities in the corresponding subspaces. The system is applicable to any subspace clustering approach, so different approaches can be compared with each other. In the work by Tatu et al. [26], 2D projections of the data in alternative subspaces were applied to identify complementary, orthogonal, or redundant subspaces; again, the approach was applicable to different subspace selection methods. In another approach [27], visual comparison of data groups across dimensions using linked views in an encompassing system was presented.

### Visualization in bio-medicine and health and distinction of our approach

To date, there are numerous approaches to tackle problems in bio-medicine and health using visual-interactive approaches. Several dedicated academic venues address the topic including, for example, the Symposium on Biological Data Visualization and the EG Workshop on Visual Computing for Biomedicine. The proposed solutions support questions from general analysis of research data and data exploration, to specific applications like health data record visualization [28] or the detection of adverse drug reactions [29]. So far, also several design challenges have been conducted to arrive at useful results^1^ [30].

In our own previous work [31], we have discussed a workflow for analysis of biomedical data based on subspace clustering analysis. The workflow was supported by tabular views to compare clusters and relationships to dimensions. Here, we substantially extend the visual analysis by an encompassing interactive system and extended workflow based on it. Our system builds on previous work in interactive visual data analysis in general and visual subspace analysis in particular. Our contribution is to present a highly interactive analysis system combining subspace analysis methods with appropriate linked views for data exploration and navigation. While we focus on a specific problem (vaccination analysis), the proposed workflow is generalizable to many biomedical data analysis questions involving labeled data. Our experimental use case and derived workflow suggests how subspace analysis methods can be leveraged, avoiding some of their practical pitfalls, e.g., generation of an abundant number of similar or redundant subspaces.

## SubVIS—interactive tool to visually explore subspaces

In the following section, we introduce SubVIS, an interactive tool which allows the user to visually analyze, explore, and interactively refine computed subspaces to gain knowledge about cluster structures in different subspaces. In the following, we are speaking of a **subspace** when describing a subset of dimensions; a **subspace cluster** or **cluster** when referring to a *single cluster* within a subspace; and **subspace cluster** **ing** or **cluster** **ing** when describing the structure of *multiple subspace clusters* as an outcome of a subspace analysis algorithm (see below).

SubVIS tackles the aforementioned challenges of subspace clustering algorithms, namely (1) difficult interpretation of the results, (2) redundancy of detected subspaces and clusters, and (3) different clustering results for different parameter settings. In contrast to tools like VISA [21], which are focusing on a global comparison of subspace clustering results (same clustering *structure* w.r.t. cluster members and subspaces), SubVIS enables the user to compare individual clusters that are detected by any subspace clustering algorithm. To this end, SubVIS analyzes every subspace cluster independent of its association to a specific clustering structure or algorithm. SubVIS is implemented as a web-based application using the d3.js^2^ JavaScript visualization library. The tool uses the detected subspaces of the *OpenSubspace Framework* [32], allowing the use of every subspace clustering algorithm which is provided by the framework. The OpenSubspace Framework is a Java Plugin for the WEKA Data Mining Library [33]. So far, we parse the detected clusters with the associated subspaces, but SubVIS can easily be extended towards a server–client architecture which can be directly plugged into OpenSubspace.

**Fig. 3:**
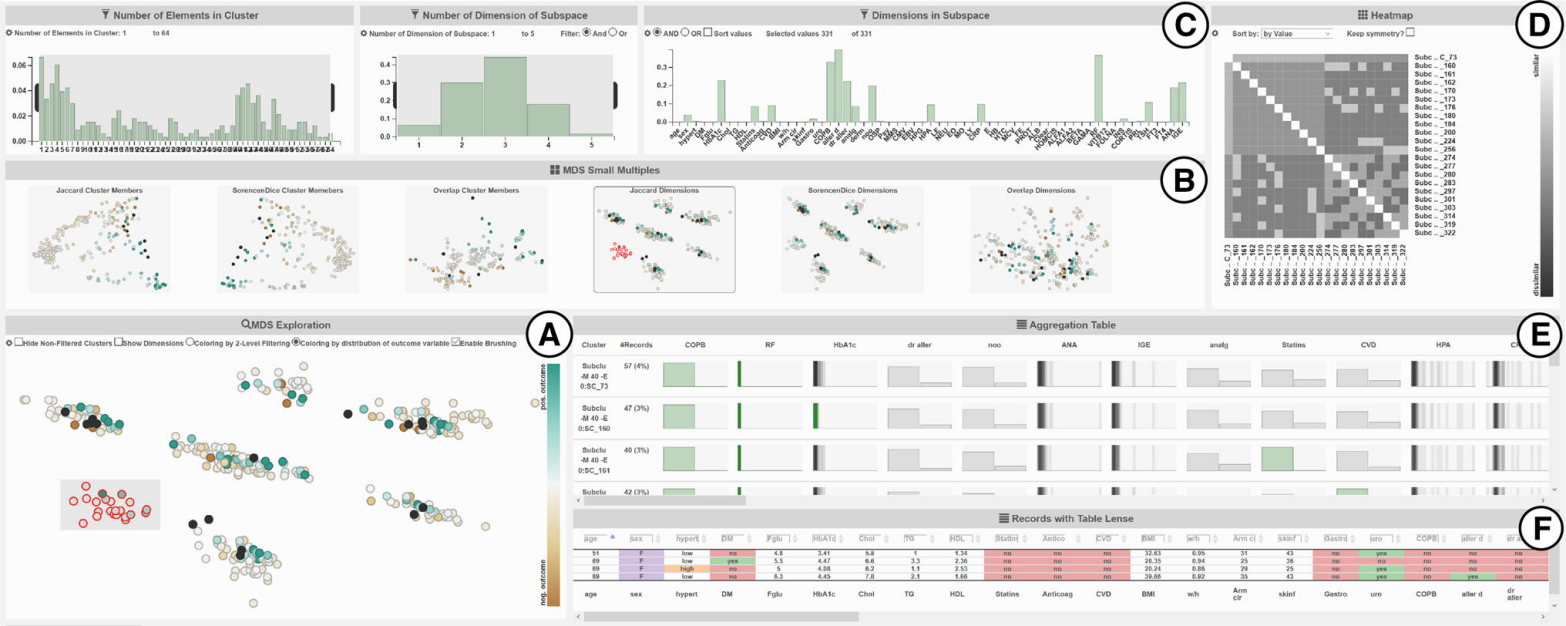
A screenshot of our visual analytics tool SubVIS. It enables the user to interactively explore a large number of subspace clusters. A general overview of the similarities between the subspaces is given by an MDS projection (**a**). Small multiples (**b**) allow to preview projections of different distance functions and a quick change of the MDS plot. On the very top (**c**), the user is provided with some distribution properties of the subspaces such as the #dimensions. A heatmap (**d**) provides more details on the relationships between the pair-wise distances. An aggregation table (**e**) shows the values of the aggregated cluster members and the table lens (**f**) provides details on demand. (Color figure online)

The general concept of SubVIS is a three-level exploration strategy based on Shneiderman’s visual information-seeking mantra [34] *Overview first, zoom and filter, then details-on-demand*. The individual visualization widgets are combined by *linking-and-brushing* and can be panned and resized based on the users’ needs. A screenshot of the tool can be found in Fig. 3.

The first exploration level of SubVIS enables the user to get a general overview of all detected subspace clusters and their properties such as the cluster and subspace size, the distribution of dimensions within the different subspace clusters as well as the similarity between them. The properties are visualized with interactive barcharts and the overview of the similarities is given by an MDS projection of the clusters combined with a matrix-based heatmap for more details. In the second exploration level, the user can select a subset of relevant clusters in the MDS plot or with the help of the barcharts and get an aggregated overview of the cluster members in a so-called *aggregation table*. For each cluster, the user is able to inspect the distribution of the cluster members in each dimension and can compare the distribution with the global distribution considering all data records. In the last exploration level, the user is facilitated with a *table lens*-like view [35] enabling the user to explore the actual data records. The table offers interactive sorting and coloring of the record and its dimension, as well as a zooming lens for more details on demand.

In the following, we describe the different visual components of the tool in more detail and emphasize on their interaction techniques. Section 4 shows the concrete usage of SubVIS with the help of a use case from the medical domain. On our website,^3^ we provide supplementary material to this paper including videos illustrating the usage of SubVIS and details about the use case described in the next section.

### General overview: MDS projections

The general overview of the subspace clusters is given as an MDS Projection as shown in Fig. 3 ①. Each dot represents a single cluster. The distance between the clusters in the MDS plot represents their pair-wise similarity (c.f. Sect. 2 for a detailed description). SubVIS contains a variety of different similarity measures which are applied on either the dimensions, or the members of a subspace cluster. Besides an Overlapping and Dice Coefficient, SubVIS includes the Jaccard Index as a similarity measure. The Jaccard Index between the dimensions of the subspaces $sub_i$ and $sub_j$ is computed as follows:

$${\rm sim}({\rm sub}_i, {\rm sub}_j) = 1 - \frac{|{\rm dim}({\rm sub}_i) \cap {\rm dim}({\rm sub}_j)|}{| {\rm dim}({\rm sub}_i) \cup {\rm dim}({\rm sub}_j) |},$$

where ${\rm dim}({\rm sub}_i)$ indicates the set of dimensions of the subspace *i*. Intuitively speaking, the Jaccard index measures the number of common dimensions in two subspaces. More advanced distance measures as introduced in [21] which are based on the combination of dimensions and cluster members can be added to the tool easily. The user can interactively change the distance measure. To do that, small multiples as shown in Fig. 3 ① are provided, which helps compare the different projections.

The coloring of the clusters in the MDS projection can be changed by the user according to the number of dimensions or cluster members, the compactness of the cluster member, or according to the distribution of a specific dimension which the user is interested in. An application for this coloring option is given in Sect. 4 where the user is interested in subspaces that contain clusters of patients for which a vaccination has been considered successful or not.

**Fig. 4:**
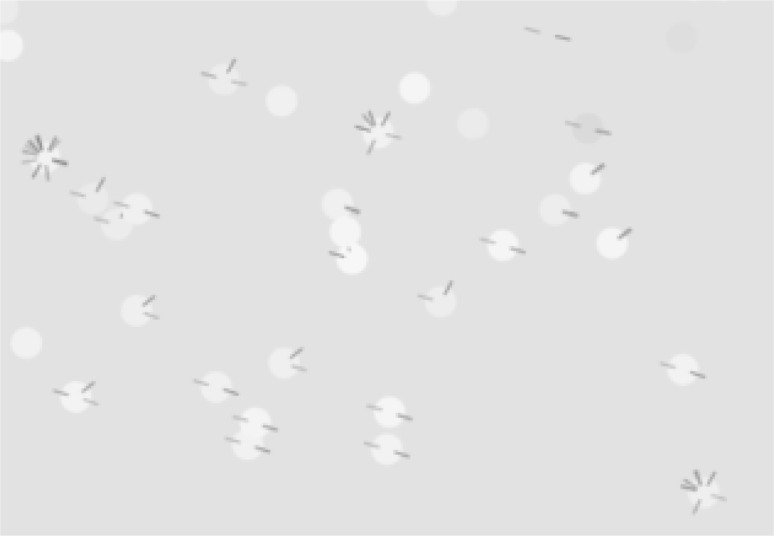
Detailed view of the subspace clusters within the MDS Plot. Each glyph represents a subspace cluster, where the involved dimensions are shown by spikes as in ClustNails [25]. In our system, we also use coloring to visually distinguish the dimensions. (Color figure online)

The user can enhance the dot representation of the clusters by a more advanced glyph representation which shows the underlying dimensions of the subspace. This representation is shown in Fig. 4 and is based on the idea of ClustNails [25]. Each dimension is represented by a small line around the border of the dot. The length of the dimension spikes can be mapped to different measures such as the importance of a specific dimension for the respective subspace. Lines are positioned according to the ordering of dimensions in the input dataset. In addition to the position, the dimensions are colored by different colors, each 10th dimension having the same color. This coloring option improves the visual distinctiveness of the dimension glyph and helps the user in finding commonalities in different clusters.

Finally, the user is able to select a single or multiple clusters by mouse to gain more insight into the cluster members and their subspaces as described below.

### Subspace filtering and recomputation

On the very top of SubVIS, the user can investigate different properties of the (selected) subspace clusters by means of interactive barcharts (c.f. Fig. 3 ①). These charts show the distribution of the subspace and cluster sizes of the selected clusters in the MDS projection. The chart on the very right side shows the occurrence and distribution of the dimensions across all selected subspaces. The user is able to filter clusters by selecting or un-selecting specific dimensions, or by changing the ranges for the #dimensions or #cluster members per cluster using a brushing technique.

The different filters are linked with each other and the MDS projection, meaning that changing one filter updates the distribution in the other filters (and the MDS projection) according to the current filter set. It can also be filtered multiple times. Subspace clusters, which are not in the filter, can be marked in very light gray or could also be removed entirely from the visualization. Both *AND* and *OR* operations can be used to link different filters and different filter settings for one filter. Moreover, further filters can be added easily.

Finally, the user is able to select a subset of dimensions and re-run the subspace clustering algorithm. This allows an analysis of more local subspace patterns which are further discussed by our workflow in Sect. 4.4.

### Heatmap

The heatmap, as shown in Fig. 3 ①, is one possibility to show more details of a selection of subspace clusters in the MDS projection. Each row and each column represent one of the selected clusters. Each cell, as a combination of two clusters, represents the similarity or dissimilarity between two clusters by means of color. Darker colors indicate dissimilarity, and brighter colors similarity. Moreover, the heatmap can be sorted to ease the perception of patterns. The advantage of the heatmap compared to the MDS representation is that a user can perceive all pair-wise similarities between all selected clusters. The MDS projection, however, can distort the perception of similarities as in many scenarios there is no optimal 2D representation of all pair-wise similarities which results in perceivable patterns which are not given in the underlying data.

### Aggregation table

The aggregation table, as shown in Fig. 3 ①, supports the user in getting an overview of the subspaces and the distribution of the attribute values of all cluster members of a small set of selected clusters. Each row in the table represents a subspace cluster, and each column a dimension. The dimensions are ordered according to their number of occurrences in the selected subspace clusters within the MDS projection. The dimensions of the subspace clusters are highlighted in green. Each cell of the table illustrates the aggregated data records within each cluster. Categorical dimensions are visualized by a histogram, while numerical dimensions are visualized by a “bar-code” whereby a dark color indicates a high occurrence and a bright color indicating a low occurrence.

The user is able to move the mouse over a specific cell and compare the distribution of the cluster members within this dimension with the global distribution considering all records of the dataset. As part of future work, we want to highlight the dimensions within a subspace for which the distribution significantly differs compared to the global distribution.

### Table lens

The user is able to investigate the data records of a single subspace cluster by means of a table lens [35] which is shown in Fig. 3 ①. Hereby, each row in the table represents a single data record, with each column, again, a dimension, and the cells contain the actual data values. The user is able to sort the table according to the different dimensions and color the cells of individual dimensions according to its values. For example, every category of a dimension is represented by a specific color, or the upper and lower quartiles or outliers of a dimension are highlighted. This highlighting gives the user a means to quickly understand the structure of the data. Typically, the zoom of the table is selected rather small, so that the user can concentrate on the coloring of the table. Additionally, the user is equipped with a lens to dynamically zoom into specific data records without changing the zoom of the whole table.

## Use case: explanations for vaccination outcomes based on subspace similarities

We study the potential of a subspace clustering-based analysis using SubVIS on a real-world medical analysis problem. We introduce a relevant dataset from clinical research, describe our analysis goals, present results of initial experiments, and interpret them from the domain perspective. The domain knowledge-based interpretation of our results is done by our co-author Majnaric who is a medical physician and researcher. Furthermore, we show the potential of the human-in-the-loop with the help of SubVIS and introduce a novel workflow for interactive subspace computation.

### Considered dataset and analysis goals

#### Dataset: vaccination_outcome

The examined dataset is based on a real-world patient dataset which describes volunteers vaccinated against influenza. The vaccination took place in a family practice located in Osijek, Croatia, during 2003/2004. Patients were selected to represent a high-risk population for influenza complications. All subjects were suffering of multiple (age-related) chronic medical conditions which interfere with the immune system. The investigated group of patients consists of 35 male and 58 female persons aged between 50 and 89 years. The dataset contains 61 dimensions describing clinical parameters such as sex, age, anthropometric measures, and hematological and biochemical tests. In addition to that, dimensions containing diagnosis results of common chronic diseases are included.

The used influenza vaccination was a trivalent inactivated split vaccine produced by Solway. It contained three seasonal virus strains: A/H1N1/New Caledonia/ 20/99-like, A/H3H2/Moscow/10/99-like, and B/Hong Kong-330/2001-like. For each strain, the vaccination was considered successful, when the amount of antibody titers was four times higher afterwards. For validation, blood samples had been taken three times prior and once four weeks after the vaccination [36]. For our analysis, we concentrated on a single target attribute representing the outcome of the vaccination for the strain A/H1N1/New Caledonia/20/99-like. From the dataset, 36 patients with a positive and 57 with a negative outcome can be identified. Further details about the dataset and the underlying influenza vaccination can be found in [37]. A list with the attributes of the dataset is available in the supplementary material of this paper.

#### Analysis perspectives

The goal of our analysis is to find reasons for a positive or negative vaccination outcome. According to our domain expert (see above), who also collected the data, the reasons for a particular outcome can be described neither by a single dimension, nor by a fixed combination of dimensions. Instead, a variety of different reasons may cause the positive or negative outcome. Therefore, global patterns such as derived by full-space clustering may not be appropriate for this kind of analysis (c.f. experiments in Sect. 4.2). As a consequence, we rather search for *local clusters* in a subset of patients and, more importantly, in a subset of dimensions. This procedure enables the analyst to find multiple, independent explanations for a possible vaccination outcome. The considered dimensions of a subspace cluster, together with its values within the cluster, can be interpreted as possible explanations for an outcome.

As mentioned before, the major challenge of a subspace cluster analysis is the interpretation of the results in conjunction with respective domain knowledge of the analyst (medical doctor in our case). Therefore, SubVIS is used to support the analyst during the exploration of the subspaces.

#### Data preprocessing

As shown above, the considered dataset is heterogeneous as it contains both numerical and categorical dimensions. Existing subspace clustering algorithms typically work on numerical data only. Furthermore, for existing subspace clustering implementations, we typically did not find recommendation as to how missing values should be treated. Taking a pragmatic approach, we preprocessed the dataset in the following way: (1) We removed all patient records that have a missing value in any of its dimensions. Afterwards, the resulting dataset contains 29 patients with a positive and 42 patients with a negative outcome. (2) We transformed all nominal dimensions such as *sex*, *hypert*, or *statins* into a numerical representation. Due to the fact that all nominal dimensions (except for diabetes mellitus (*DM*)) consist of only two different values (mainly *yes* and *no*), we converted the values to either 0 or 1. Finally, we normalized all dimensions linearly in the range [0,1] according to the following formula:

$${\text{normalized}}\;{\text{value}}_{j}^{{\rm dim}_i} = \frac{{\rm value}_j^{{\rm dim}_i} - {\rm min}({\rm dim}_i)}{{\rm max}({\rm dim}_i) - {\rm min}({\rm dim}_i)}.$$

After this, all dimensions are numerical in the range of [0,1], enabling further analysis with equally weighted dimensions.

### Experiments in full-space analysis

In our initial experiments on the dataset, we first confirmed that a full-space analysis is not useful. We used data mining tools such as KNIME [38] and WEKA [33] to cluster patients into different groups, or applied different classification algorithms to correctly predict the vaccination outcome of a patient. The details are discussed in the following.

**Fig. 5:**
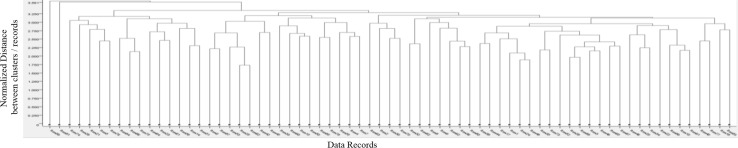
Dendrogram illustrating the hierarchical (full-space) clustering of our vaccination dataset (*Euclidean distance* and *average linkage* type). The x-axis represents the individual patients, while the y-axis indicates the (dis-)similarity between two patients or a cluster of patients. A large y-value corresponds to a large dissimilarity

#### Full-space Experiment 1: clustering

A hierarchical full-space clustering was applied. The results are illustrated as a dendrogram in Fig. 5. The x-axis is mapped to the individual data records (patients) and the y-axis represents the similarity between two patients or clusters of patients. A low y-value indicates a high similarity, while a large y-value corresponds to a high dissimilarity between the underlying patients. From Fig. 5, we see that none of the patients are considered similar as all “splits” are high up in the diagram. As a consequence, no useful grouping of patients can be identified. We assume the following reasons: (1) patients are typically similar to each other only in a subset of dimensions; (2) a similarity in one dimension can be countered by a dissimilarity in another dimension; and (3) the *concentration effect* [3] affects the similarity computation in high-dimensional spaces.

#### Full-space experiment 2: classification

For the classification task, we did not remove missing values but rather replace them by the average value of the dimension. We applied several classification algorithms to find useful predictors for the vaccination outcomes. Our experiments comprised, for example, *Decision-Trees*, *Bayes Classification*, and *Random Forest*. We split the dataset into a training set (80 % of the records) and a validation set (20 % of the records). For the validation, we measured the percentage of correctly classified patients after the model training. While the accuracy of the classification of the training dataset is very well (approx. 84 % for decision tree), the accuracy for the validation dataset dropped below 50 % for some algorithms, which is worse than that for random classification. We assume that the poor classification performance is caused by (1) the size of the training dataset which is too small, and (2) there are no global aspects allowing a classification into the two outcome classes. Instead, different combinations of features may be of relevance to predict the outcome properly.

### Subspace analysis: initial experiments and results

To search for local explanations of the vaccination outcome, subspace analysis techniques are beneficial. In the following, we describe three different experiments that we conducted using SubVIS. The experiments apply the subspace clustering algorithm *Proclus* to different subsets of the data. The discovered subspaces are then used as a means to describe the similarity between subsets of patients with either a positive or negative outcome. The dimensions of the subspace cluster describe in which way the patients of a cluster are similar to each other which may also indicate as a reason for a possible vaccination outcome. For a better understanding of the different experiments, we provide a video^4^ with a detailed description of our analysis.

#### Background: Proclus

For our experiments, we rely on a subspace clustering approach called *Proclus* (Projected Clustering) [39]. Proclus is similar to *k-means* [10] as it generates, by an iterative process, a *partition* of the data. Each data point can belong to one cluster, and each cluster is represented by a prototype point (medoid). Proclus needs two parameters: number of clusters *C* and average dimensionality per subspace *D*. The subspace computation starts by a random initialization of medoids. In a refinement step, for each of the *C* medoids a well-fitting subspace of average dimensionality *D* is found. This is achieved by finding dimensions that show a low variance of the distances between the respective medoid and its cluster members. The resulting subspace contains dimensions in which the values of the cluster members are similar. While other subspace clustering methods are available [14], we chose Proclus for its simplicity, efficiency, and robustness to noise, using the *OpenSubspace Framework* [32] implementation.

#### Subspace experiment 1: combined outcome

**Fig. 6:**
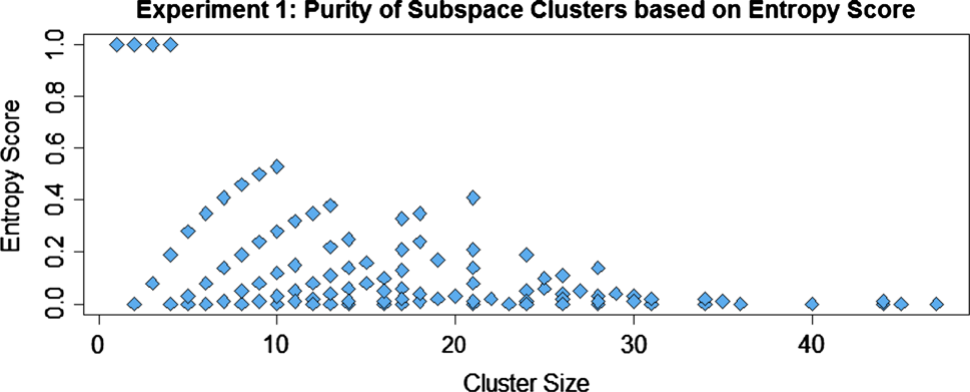
Measuring the purity of subspace clusters w.r.t. the vaccination outcome. Overplotting occurred due to identical combination of the #clusters and entropy score

**Fig. 7:**
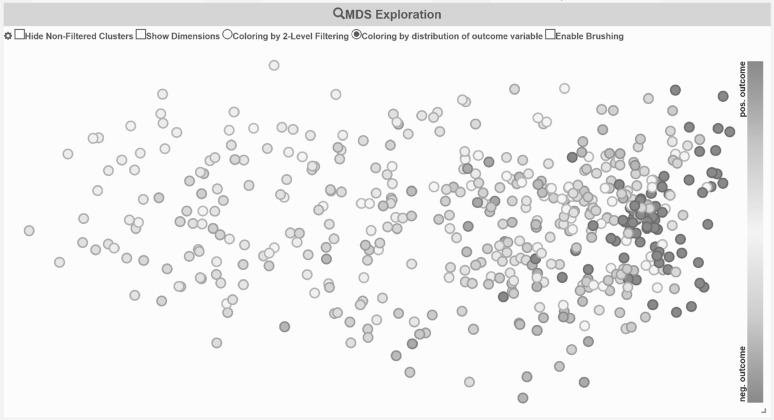
MDS projection of the subspace clusters which are detected by Proclus. The Jaccard distance between the cluster members has been selected. Both intense blue and brown colors represent clusters containing mainly patients of one outcome class, while a bright color indicates that a cluster contains patients of both classes. (Color figure online)

In the first experiment, we apply Proclus to the preprocessed dataset and aim for subspace clusters that contain mainly patients of a single outcome. If not mentioned otherwise, we vary Proclus’ parameters #clusters: 2–8 and avg. #dimensions: 3–14 for all of our experiments. We combine the detected subspace clusters from all runs of the Proclus algorithm into SubVIS. This combined analysis allows us to explore a large number of subspace clusters computed by different parameter settings of the underlying algorithm (instead of analyzing each run separately). Afterwards, we evaluate each detected cluster with the *Entropy score* [32] which measures the purity of a cluster w.r.t. a specified class label, i.e., the vaccination outcome. Figure 6 illustrates the distribution of the score. We can see that almost none of the detected clusters contain patients of only one specific class, but rather a mixture of both classes without a significant majority of a positive or negative outcome. This is also confirmed by SubVIS which can be seen in Fig. 7. The color in the corresponding MDS projection represents again the purity of a cluster w.r.t. to one outcome class. Cluster containing mainly patients with a positive outcome are represented as blue, clusters with patients of a negative vaccination outcome are represented as brown, and clusters with a mixture of both outcomes are illustrated with a very bright color. Using this color scheme, the user can concentrate on the clusters with an intensive color, i.e., homogeneous patients. We believe that the result is caused by (1) the computation strategy of Proclus which aims for large clusters, and (2) the dataset contains dimensions in which many patients are similar to each other—independent of their class label. These dimensions dominate the detected clusters and prevent Proclus from finding clusters relevant for the description of the vaccination outcome (c.f. experiment 2).

**Fig. 8:**
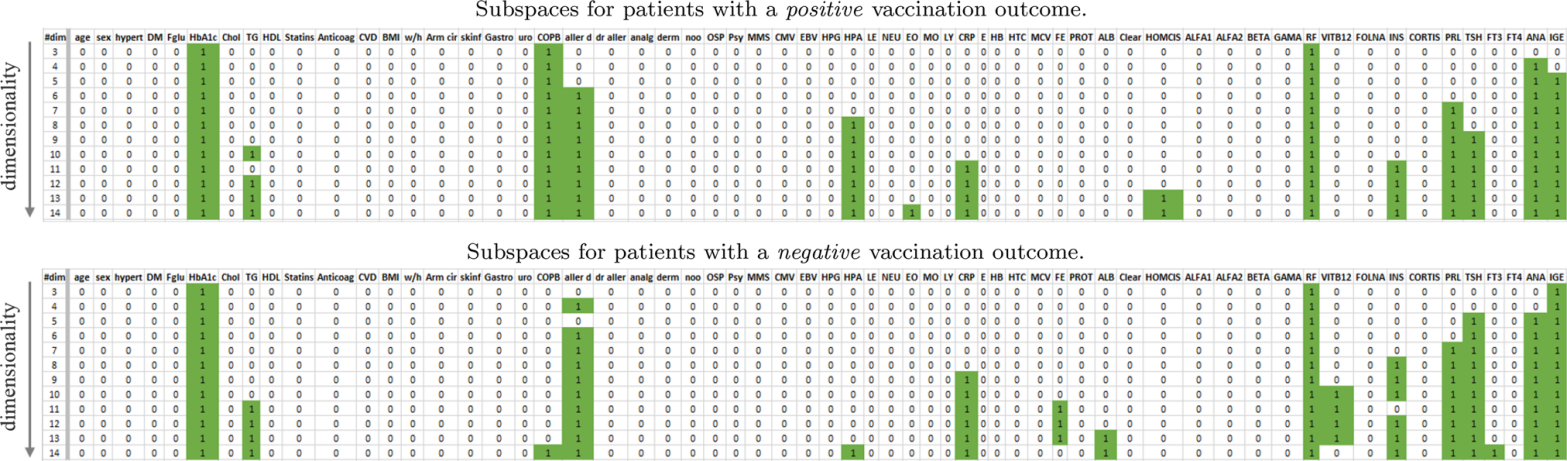
Subspaces detected by experiment 2: subspace clustering (*Proclus*) applied separately to patients with a positive or negative outcome. The *columns* represent the different dimensions (*green* indicates that the dimension belongs to subspace). Each *row* represents a clustering result of different dimensionality. (Color figure online)

#### Subspace experiment 2: separate outcome

To find descriptive clusters for each vaccination outcome, we split the dataset into subsets according to the outcome class. Afterwards, Proclus is applied to each subset separately. The resulting subspace clusters of both subsets are combined into SubVIS for a visual exploration. The detailed steps of this exploration can be found in the above-referenced video.

In the first part of the experiment, we configure *Proclus* to detect subspaces containing a *single cluster*. For each subset, the average number of dimensions again varies between 3 and 14. The results can be found in Fig. 8. The different dimensions are indicated as columns, while each row represents a subspace cluster with a different dimensionality. The cells of a row are marked with a green background, if the subspace contains the dimension, e.g., the first subspace for a positive outcome contains the dimensions: *HbA1c*, *COPB*, and *RF*.

**Fig. 9:**
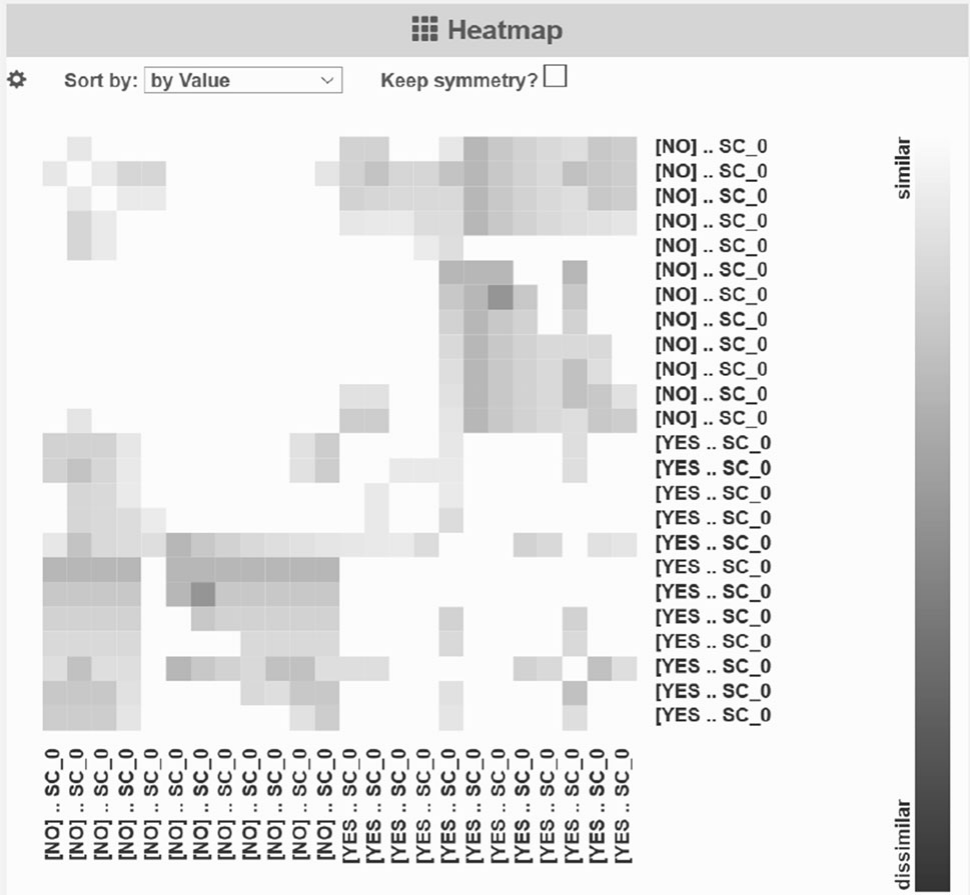
Heatmap representing the similarities of the detected subspaces of the second experiment based on the overlapping coefficient. All combinations of subspaces share a very *light gray color*, meaning that these subspaces are similar to each other

*Proclus* determines the dimensions of a subspace cluster by ordering all dimensions by the variance of its cluster members, and selecting the dimensions with a minimum variance (c.f. description of Proclus above). Therefore, subspaces with a larger dimensionality may include dimensions in which its cluster members are less similar. As all records belong to the same cluster, dimensions in lower-dimensional subspaces are more descriptive for an outcome class (w.r.t. global outcome similarity). Consequently, the height of the green bars in Fig. 8 illustrates the importance of a dimension for an outcome class. Except for *HPA* and *PRL*, the globally descriptive dimension is identical for both outcomes. We can also see in Fig. 9 of SubVIS that the detected subspaces for both outcome classes are quite similar and there are no subspaces which are specific of one outcome class. This result is in line with the detected subspaces of the first experiment, i.e., the following set of dimensions is discriminative for all patients from a global perspective: *HbA1c*, *COPB*, *aller d*, *HPA*, *CRP*, *RF*, *INS*, *PRL*, *TSH*, *ANA*, and *IGE*.

Most patients in our dataset are similar in these dimensions; however, we do not gain much knowledge about the patients w.r.t. the vaccination outcome. This observation is confirmed by the second part of experiment 2. In addition to the first part, we also varied the #clusters between 2 and 4. The complete result can be found in the supplementary material. In summary, we can see that even for results with 4 clusters, the majority of dimensions are from the given set above. From the second experiment, we can conclude that subspace clustering helps find dimensions in which patients of a specific class are similar to each other, and hence these dimensions may be an indicator for the reason of classification. However, experiment 2 shows that dimensions in which most patients are similar to each other highly influence the clustering results. As a consequence, the subspaces for both outcome classes are similar to each other.

#### Subspace experiment 3: dimension refinement

**Fig. 10:**
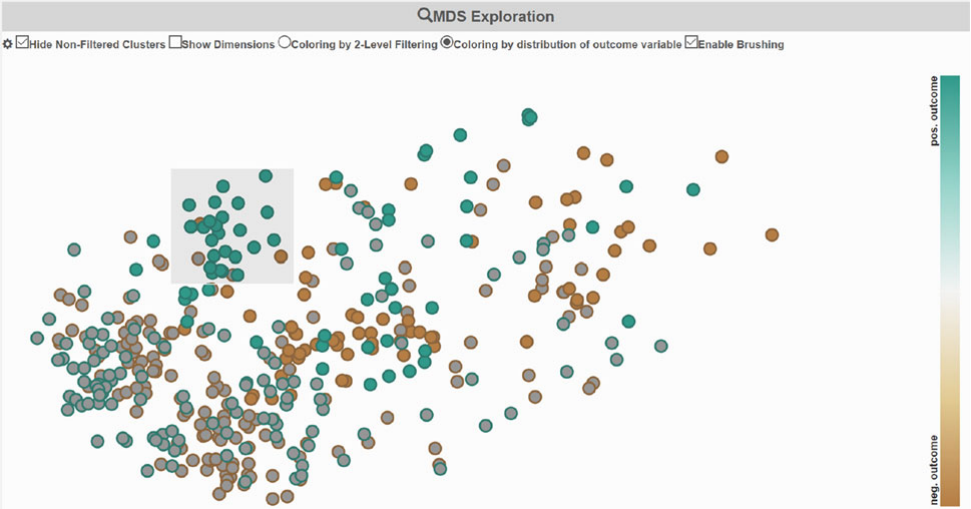
Selecting a relevant subset of subspaces which contain only patients of a positive vaccination outcome. Subspace clusters with less than 5 patients or less than 4 records are filtered out beforehand (marked as *gray*). (Color figure online)

In our last experiment, we concentrate on *more local patterns*. From the previous experiments, we know that all patients, and in particular all patients of one outcome class, are similar to each other in the dimensions described above. To find more local patterns, we remove these dimensions from both subsets and re-apply Proclus. Afterwards, we used SubVIS to seek for subspace clusters which are similar to each other and descriptive for one specific outcome class. We provided the subspaces to our domain expert and let her analyze the clusters. For the positive vaccination outcome, the user selected a group of clusters as shown in Fig. 10. To analyze negative vaccination outcomes, the domain expert focused mainly on clusters which contains the dimension *hypertension*. Therefore, we filtered for these subspaces and selected again a group of similar subspaces (as shown in the video).

The domain expert analyzed the resulting subspace clusters with the help of the aggregation table to get quickly an overview of the data distribution within the clusters and with the table lense to analyze details of the individual patients. She found patterns in the detected clusters which explain—at least to some extent—the reasons for a positive or negative vaccination outcome. In the following, we shortly describe two of her findings:

*Positive vaccination outcome* The selected subspace clusters contain mainly patients with homogeneous values in its dimensions which indicates that the patients are rather healthy. The patients do not have hypertension (*Hyper*), cardiovascular diseases (*CVD*), psychiatric disorders (*Psy*), and adverse reaction to drugs (*dr aller*). Furthermore, the patients do not use any of the following medications: statins (*Statins*), anticoagulants (*Anticoag*), or analgesics (*analg*) which results in preserved renal function (*CLEAR*).

*Negative vaccination outcome* A couple of subspace clusters show a clear reason for a negative vaccination outcome. A key discriminator, which has already been identified in earlier studies, is vitamin B12 (*VITB12*) and folic acid deficiency (*FOLNA*). The values in these dimensions can be a marker of impaired methylation reaction, indicating impaired epigenetics and DNA instability. This condition can be associated with impaired proliferation and division of lymphocytes, which is a key element for the development of the immune reaction. These aspects can be seen as a possible link between vitamin B12 and folic acid deficiency and the low antibody response after an influenza vaccination, i.e., a negative vaccination outcome.

### Proposed subspace analysis workflow

**Fig. 11:**
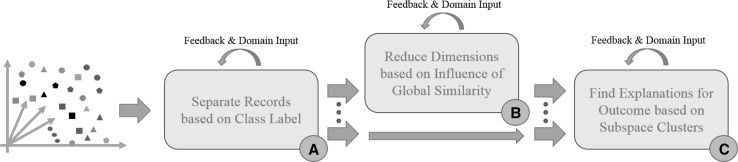
Our proposed workflow to discover relations between patients, relevant dimensions, and different class labels (here vaccination outcomes). (Color figure online)

In general, the appropriate application of a subspace clustering algorithm (e.g., Proclus) is often found to be more complex than applying a comparable full-dimension clustering algorithm (e.g., k-means). This is due to the increased number of parameters and complexity of the resulting output of subspace clustering. The detected clusters are bound to an underlying subspace and often many redundant clusters are found. SubVIS helps leverage the added complexity by interpreting the results and give the user the possibility to explore the detected subspaces from different perspectives. Furthermore, the domain expert can inject his/her domain knowledge by filtering for specific subspaces. Based on our findings in the experiments described above, we propose a subspace clustering-based workflow (c.f. Fig. 11) to find relations between data records, dimensions, and associated class labels. The workflow consists of the main steps ① and ① as well as an optional step ① improving local similarity aspects as shown in the third experiment. The workflow described below is fully supported by SubVIS.

For class-labeled data, the first step of the workflow is to separate all data records based on their class label ①. For non-class-labeled data, the first step can be discarded. The subsequent steps are applied to each record subset individually. The optional step ① is in line with the findings of the second experiment. In many datasets, there are dimensions that highly influence the detection of subspace clusters. On the one hand, these dimensions are interesting as they show the global similarity between data records. On the other hand, such dimensions can distort the results, e.g., a dataset with non-relevant dimensions in which all records are similar. Subspace clustering considers these dimensions as relevant and adds them to most clusters. In such a case, step ① can be applied to remove such dimensions. In ①, a subspace clustering is applied to the remaining dimensions to finally determine the similarities between records, dimensions, and class labels. In extension to our previous findings in [31], we extended the workflow to enable it with user feedback and injection of domain knowledge. We illustrated this extended workflow with the usage of SubVIS for the descriptive use case above.

## Discussion

The explorative analysis of patient treatment data is a challenging task. As our experiments show, subspace clustering can be a valuable tool to discover relevant groups of patients w.r.t. different medical subspaces and their relationship to the treatment (here vaccination outcome). As a key finding of our experiments, an analysis in the full attribute space may not be the best choice, but subspace methods can be an interesting tool, especially if used in an appropriate analysis workflow. We proposed one workflow, considered as a promising starting point.

We also identify a number of extension possibilities to our approach. For one, we may need heuristic criteria which could select from a large number of parameters (e.g., input dimensions, number of clusters, distance thresholds, etc.) a small number of results which are not redundant but can be meaningfully interpreted. To this end, we need a formalization of how to measure what alternative or complementary means in terms of dimensions, cluster size, and attribute subsets. We need to include additional medical background into such a specification. Visual interfaces may be particularly beneficial to this end. A key issue in visualization is how to effectively map patient records, cluster, and attribute properties to visual displays. Regarding data size, scalability of the cluster analysis may become an issue, which could be addressed by efficient implementations.

We employed Proclus which considers all dimensions of a subspace as equally important for the subspace. However, there may also exist non-linear relationships between attributes which might be relevant. Alternative analysis tools like non-linear multivariate regression could be considered to optimize attribute selection. Also on the preprocessing side, how to appropriately treat categorical and binary attributes in the analysis is a problem. We here choose standard approaches, but the expert may need to specify how to treat such attributes.

While analysis is often handled by ad hoc approaches, it would be desirable to have a software framework to allow a flexible, interactive specification of analysis workflows, to easily apply and re-use proven workflows. We envision a workflow editor which could support the analysis process in a scalable way and, at the same time, enable experts to document which and why analysis steps were taken.

## Conclusion and future outlook

The life sciences, bio-medicine, and health care are turning into a data-intensive science, where we face not only increased volumes and a diversity of highly complex, multidimensional, and often weakly structured and noisy data, but also the growing need for integrative analysis and modeling [1]. Considering that analysis in the full attribute (feature) space may not be effective, we here explored subspace cluster analysis to study the relationship between patient data and immunization treatment outcome on a specific research dataset. We found that a segmentation of the patients for treatment outcome followed by subspace clustering allowed the identification of relevant patient groups and respective medical attributes, which can be a basis to generalize medical knowledge. Our proposed workflow is only a first step, and we identified a number of interesting challenges and extensions for future work in the area. The grand vision for the future is to effectively support human learning with machine learning—visualization is close to the end-user, hence indispensable within this approach [40]. Discovering knowledge in such complex, high-dimensional datasets needs a concerted effort of various topics, ranging from data preprocessing, data fusion, data integration, and data mapping to interactive visualization within a low-dimensional space [41]. Visual exploration methods, e.g., visual data mining [42], play a particularly important role and the adequate selection of visualization techniques finally decides about the overall success of applications for the biomedical domain [43].

## Footnotes

1 http://healthdesignchallenge.com/

2
http://d3js.org/

3 Website: http://files.dbvis.de/brin2016 Video: http://files.dbvis.de/brin2016#video

4
http://files.dbvis.de/brin2016#video_experiments
